# Improved identification of left atrial enlargement in patients with obesity

**DOI:** 10.1007/s10554-023-02981-0

**Published:** 2023-10-26

**Authors:** Yaar Aga, Yalin Acardag, Jie Fen Chin, Daan Kroon, Sanne Marjolein Snelder, Lotte De Groot-De Laat, Ulas Biter, Felix Zijlstra, Jasper Brugts, Bas van Dalen

**Affiliations:** 1 Department of Cardiology, Franciscus Gasthuis & Vlietland, Kleiweg 500, Rotterdam, 3045 PM The Netherlands; 2 Department of Cardiology, Erasmus University Medical Center, Thoraxcenter, Rotterdam, The Netherlands; 3 Department of Cardiology, Maasstad Hospital, Rotterdam, The Netherlands; 4 Department of Bariatric Surgery, Franciscus Gasthuis & Vlietland, Rotterdam, The Netherlands

**Keywords:** Obesity, Diastolic dysfunction, HFpEF, Left atrial strain, Left atrial volume

## Abstract

Accurate standardization of left atrium volume (LAV) in patients with obesity is challenging. The aim of this study was to investigate and to examine the relation between LAV indexed to height^2^ and left atrial function in patients with moderate to severe obesity. Echocardiograms of patients with moderate to severe obesity (body mass index (BMI) ≥ 35 kg/m^2^) without known cardiac disease were analyzed. LAV was indexed to body surface area (BSA) and height^2^, and patients were divided into those with or without left atrial enlargement (LAE) based on normalization using either BSA (LAE_bsa_) or height^2^ (LAE_h2_). Using speckle tracking echocardiography, LA reservoir strain (LASr), LA conduit strain (LAScd), and LA contractile strain (LASct) were assessed as a measure of LA function. LA dysfunction was defined as LASct < 14%. A total of 142 patients were included in the analysis of whom 54.2% had LAE_h2_ and 18.3% LAE_BSA_. The LAE_h2_ group had significantly lower LASct (12.2% ± 3.2% vs. 13.6% ± 4.5%, p = 0.019) as compared to the patients without LAE_h2_. Significantly more patients with LA dysfunction would be correctly identified by LAE_h2_ than by LAE_BSA_ (41.5% vs. 15.0%, p < 0.001). In patients with moderate to severe obesity, the use of LAE_h2_ identified significantly more patients with decreased LA function. LAV_h2_ should be preferred over LAV_BSA_ in patients with moderate to severe obesity.

## Introduction

Left atrial enlargement (LAE) is well established as a prognostic marker in heart failure with preserved ejection fraction (HFpEF) and is used as one of the morphologic diagnostic criteria to diagnose HFpEF [1]. Current ESC guidelines recommend indexing LAV to body surface area (LAV_BSA_) to determine LAE because of the widely available data [2]. However, since BSA is mainly driven by an increase in fat mass, indexing LAV to BSA can lead to overcorrection of LAV among patients with obesity and thereby has the potential of normalizing LA dilatation. Moreover, LAV indexed to BSA is an isometric measure that assumes a linear relationship between LAV and BSA, which is incorrect since heart and body size do not grow proportionally [3]. This is especially relevant since the majority of heart failure patients are either overweight or have obesity [4, 5].

It has been suggested that a more appropriate measure to define LAE in patients with obesity could be to use allometric scaling by indexing LAV to height^2^ (LAV_h2_) [6]. Recent studies have demonstrated that indexing LAV to height^2^ better predicts mortality in patients with severe obesity, whereas indexing to BSA has limited predictive value in these patients [7, 8].

Another emerging parameter of the LA in obesity patients is LA strain [9]. A previous study by our group demonstrated that patients with obesity have impairment in LA function before alterations in conventional echocardiographic parameters occur [10]. The potential value of LAV_h2_ may be underscored if this parameter would be related to LA function, which has not been investigated before. Therefore, the purpose of our study was to investigate the relation between LAV_h2_ and LAS, and to further establish the added value of LAV_h2_ as a parameter for LAE in patients with moderate to severe obesity.

## Methods

For this study, echocardiograms of the CARDIOBESE study and AF OBESE study were used. The CARDIOBESE and AF OBESE study are both multicenter prospective cross-sectional studies in which 192 patients with obesity without known cardiac disease were enrolled, who were referred for bariatric surgery in the Franciscus Gasthuis & Vlietland and Maasstad Ziekenhuis, both in Rotterdam, the Netherlands [11, 12]. Patients were enrolled if they were between 35 and 65 years old. All patients had a BMI of ≥ 35 kg/m^2^. Height (in meters) and weight (in kilograms) were measured at the time of the echocardiogram. BMI was calculated as weight/height^2^. BSA was calculated by using the Du Bois formula (BSA [m^2^]) = 0.007184 x height [cm]^0.725^ x weight [kg]^0.425^. Study protocols were approved by the local ethics committee and participants provided written informed consent.

### Transthoracic echocardiography

Two-dimensional greyscale harmonic images were obtained in the left lateral decubitus position using a commercially available ultrasound system (EPIQ 7, Philips, the Netherlands), equipped with a broadband (1–5 MHz) X5-1 transducer. All acquisitions and measurements were performed according to the current guidelines [2, 13]. LAV was measured on the 4-chamber and 2-chamber view. LAV was then indexed to height^2^ (LAV_h2_) and BSA (LAV_BSA_). LAE_h2_ was defined according to the ESC/ESH hypertension guidelines (LAV_h2_ >18.5 ml/m^2^ in males and LAV_h2_ >16.5 ml/m^2^ in females) [14]. When BSA was used, LAE_BSA_ was defined as LAV_BSA_ > 34 ml/m^2^ [2]. For a sub-analysis, the study population was split by obesity class according to the World Health Organization definition to check the difference in prevalence of LAE when using LAV_h2_ and LAV_BSA_ [15].

**Fig. 1:**
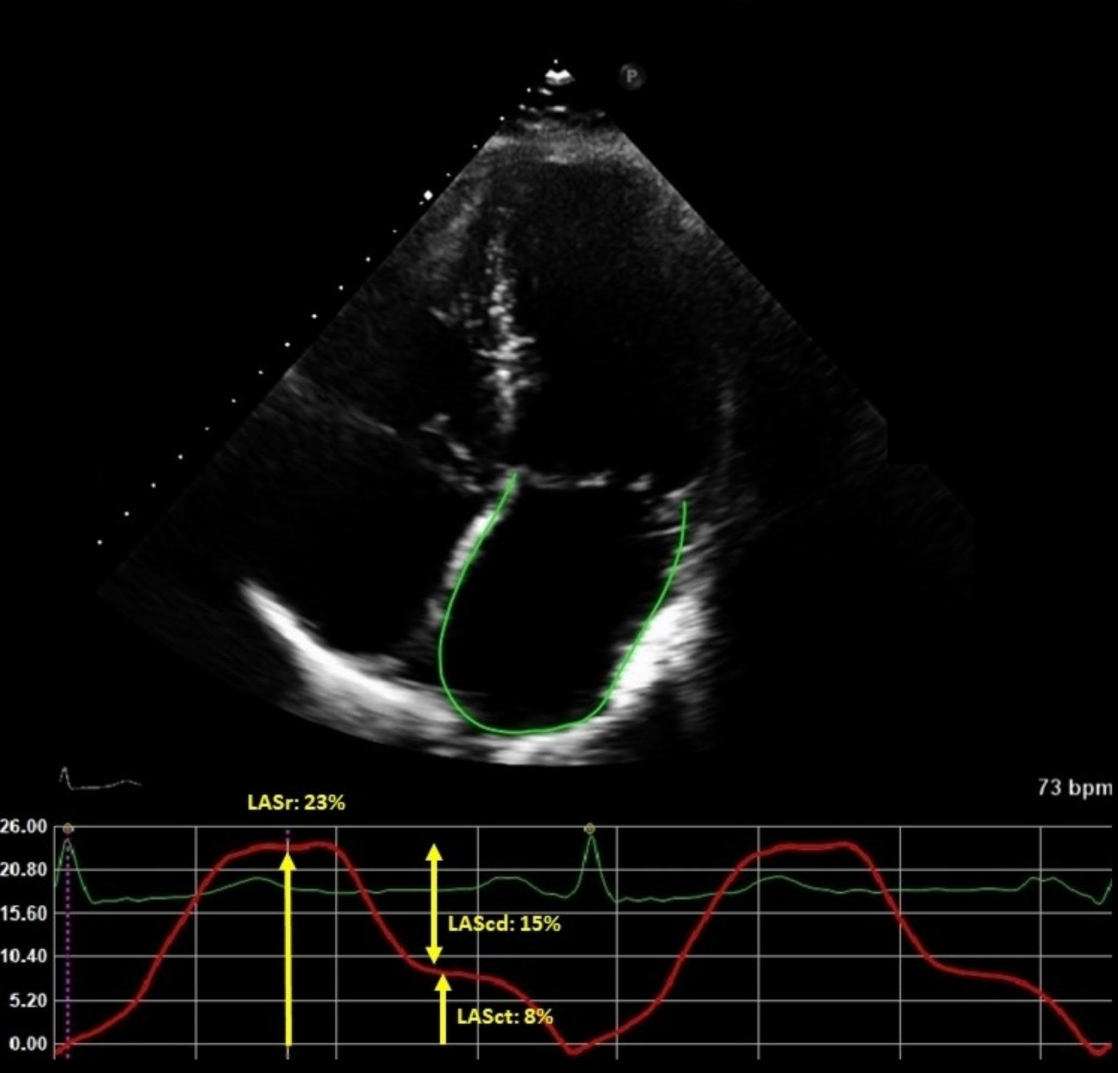
Example of LA strain curve in a patient with obesity. LASr: Left Atrial Reservoir Strain, LAScd: Left Atrial Conduit Strain, LASct: Left Atrial Contractile Strain

LA strain was measured with speckle tracking and analyzed offline with dedicated software (TomTec-Arena, integrated in Sectra IDS7). The apical 4-chamber view was used preferably for the analysis. LA endocardial borders were automatically traced using end-diastole as reference. When tracking was suboptimal, fine-tuning was performed manually. If the 4-chamber view was of poor image quality, the 2-chamber view was used. Patients with images of insufficient quality to perform LA strain analysis were excluded. LA function was described according to the three phases of the LA cycle: LA reservoir strain (LASr) which starts at the end of ventricular diastole (mitral valve closure) and continues until mitral valve opening, LA conduit strain (LAScd) which occurs from the time of mitral valve opening through diastasis until the onset of LA contraction, and LA contractile strain (LASct) which occurs from the onset of LA contraction until the end of ventricular diastole (mitral valve closure). LASr, LAScd, and LASct were computed in all patients. An example of LAS measurement in a patient with obesity is shown in Fig. 1.All strain values are reported as absolute values for improved readability and data interpretation [16]. LA dysfunction was defined as LASct < 14% [17].

### Statistical analysis

Normally distributed data are presented as means and standard deviation, skewed data as medians and inter-quartile range, and categorical variables as percentages and frequencies. Continuous variables were compared using the independent student T-test in case of normally distributed data and the Mann-Whitney U test for non-normally distributed data. Categorical data were analyzed with the Chi-square test and the McNemar’s test for respectively normally and non-normally distributed data. Statistical significance was defined as a *p* value less than 0.05. Univariable binary logistic regression (with odds ratio (OR) as main analysis) was used to assess whether abnormal LASct was associated with parameters of diastolic function. Parameters of diastolic function were dichotomized according to defined normal values [2]. Analyses were performed using SPSS Statistical Package version 28.0.

## Results

**Fig. 2:**
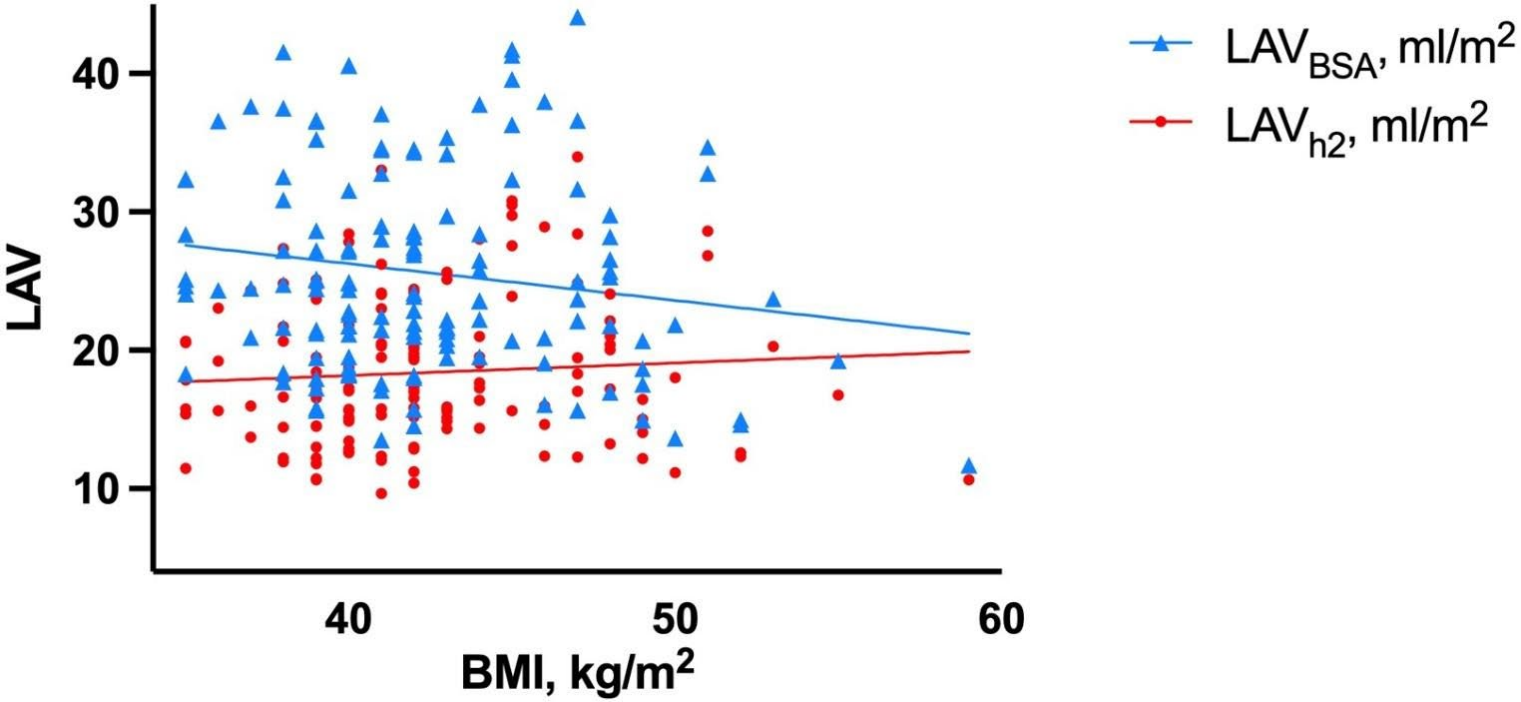
Blue triangles and line represent left atrial volume indexed to body surface area (LAV_BSA_); red dots and line left atrial volume indexed to height^2^ (LAV_h2_). BMI = body mass index

**Fig. 3:**
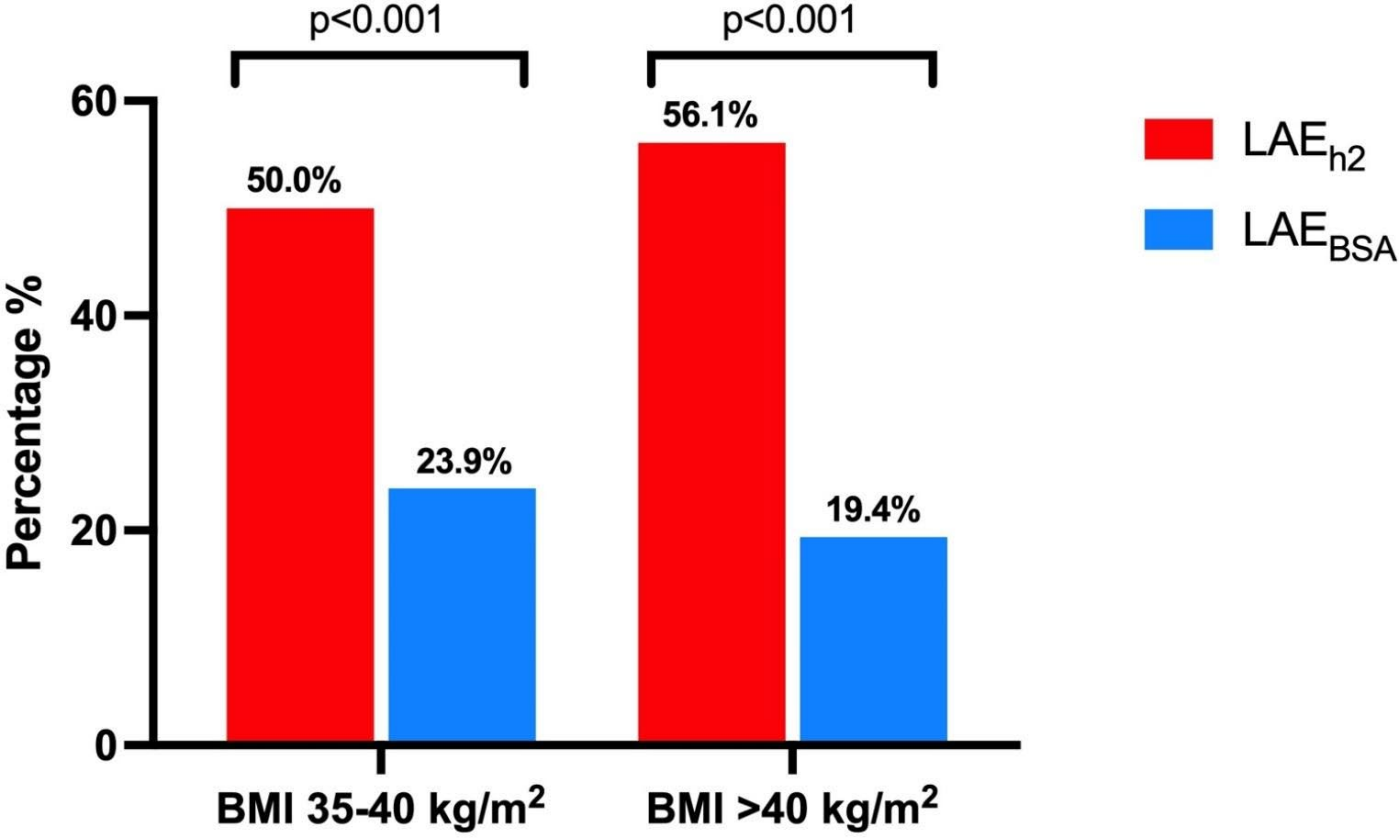
Blue bars represent left atrial enlargement indexed to body surface area threshold (LAE_BSA_); red bars left atrial enlargement indexed to height^2^ threshold (LAE_h2_). LAE_BSA_ was defined as > 34 ml/m^2^; LAE_h2_ as > 16.5 ml/m^2^ for females and > 18.5 ml/m^2^ for males. BMI = body mass index

Image quality was insufficient to quantify LA strain in 50 patients, leaving 142 patients for the analysis. Clinical characteristics of the study population are shown in Table 1. 79.6% of the patients were female. Mean age and mean BMI were respectively 52.3 ± 7.3 years and 42.4 ± 4.4 kg/m^2^. As shown in Table 2, in the total study population LAV_BSA_ was 25.6 ± 7.5 ml/m^2^ and LAV_h2_ was 18.4 ± 5.3 ml/m^2^, resulting in a total of 26 (18.3%) patients having LAE_BSA_, and 77 (54.2%) patients having LAE_h2_. In Fig. 2, LAV_BSA_ and LAV_h2_ were plotted against BMI. As can be seen, LAV_BSA_ decreased with increasing BMI, whereas LAV_h2_ increased with increasing BMI. The prevalence of LAE_h2_ was significantly higher than LAE_BSA_ in both obesity class groups (obesity class 2: p < 0.001; obesity class 3 p < 0.001) (Fig. 3). As for LA function, LASr was 30.0 ± 7.8%, LAScd 17.1 ± 6.4%, and LASct 12.8 ± 3.9% in the total study population.

**Table 1:** Clinical characteristics of the study population

	Total (n = 142)	LAE_h2_ (n = 77)	No LAE_h2_ (n = 65)	p-value
Age, *years*	52.3 ± 7.3	51.1 ± 7.9	53.7 ± 6.3	0.033
Female, *n (%)*	113 (79.6)	63 (81.8)	50 (76.9)	0.471
Weight, *kg*	121.0 ± 17.8	121.7 ± 16.9	120.1 ± 19.0	0.592
Height, *m*	1.69 ± 0.09	1.69 ± 0.1	1.68 ± 0.1	0.647
BMI, *kg/m*^*2*^	42.4 ± 4.4	42.5 ± 4.2	42.2 ± 4.6	0.704
Systolic BP, *mmHg*	146.0 ± 21.4	147.7 ± 24.0	144.0 ± 17.8	0.317
Diastolic BP, *mmHg*	79.7 ± 11.0	79.6 ± 12.1	79.7 ± 10.1	0.971
Heartrate, *bpm*	79 ± 13	76 ± 12	82 ± 12	0.007
Diabetes mellitus, *n (%)*	26 (18.4)	14 (18.2)	12 (18.8)	0.931
Hypertension, *n (%)*	49 (34.5)	33 (42.9)	16 (24.6)	0.023
OSAS, *n (%)*	28 (19.7)	16 (20.8)	12 (18.5)	0.729
Beta-blocker, *n (%)*	16 (11.3)	11 (14.3)	5 (7.7)	0.216
ACE-inhibitor, *n (%)*	18 (12.7)	10 (13.0)	8 (12.3)	0.904
ARB, *n (%)*	18 (12.7)	12 (15.6)	6 (9.2)	0.257
Diuretics, *n (%)*	27 (19.0)	20 (26.0)	7 (10.8)	0.021

LAE_h2_, left atrial enlargement indexed to height^2^; BMI, body mass index; BP, blood pressure; bpm, beats per minute; OSAS, obstructive sleep apnea syndrome; ACE, angiotensin converting enzyme; ARB, angiotensin receptor blocker. LAE_h2_ was defined as > 16.5 ml/m^2^ for females and > 18.5 ml/m^2^ for males. Normally distributed data are presented as mean ± sd, non-normally distributed data are presented as median (25th interquartile – 75th interquartile), categorical data are presented as n (%). P-value represents comparison between LAE_h2_ and No LAE_h2_

**Table 2:** Echocardiographic parameters of the study population

	Total (n = 142)	LAE_h2_ (n = 77)	No LAE_h2_ (n = 65)	p-value
LVEDD, *mm*	48.1 ± 6.1	49.4 ± 6.0	46.6 ± 5.9	0.004
E/A ratio	1.0 ± 0.3	1.0 ± 0.3	0.9 ± 0.2	0.241
E/e’ ratio	9.6 ± 2.8	9.6 ± 3.0	9.6 ± 2.5	0.902
Septal e’ velocity, *cm/s*	7.6 ± 1.9	7.8 ± 1.8	7.4 ± 1.9	0.254
TR velocity, *m/s*	1.14 (0.9–1.81)	1.13 (0.86–1.48)	1.29 (0.92–2.09)	0.112
LAV, *ml*	52.8 ± 17.8	63.9 ± 16.4	39.7 ± 7.3	< 0.001
LAV_BSA_, *ml/m*^*2*^	25.6 ± 7.5	30.8 ± 6.0	19.6 ± 3.1	< 0.001
LAV_h2_, *ml/m*^*2*^	18.4 ± 5.3	22.1 ± 4.3	13.9 ± 1.9	< 0.001
LVEF, *%*	57.2 ± 5.6	57.7 ± 5.8	56.6 ± 5.4	0.274
LASr, *%*	30.0 ± 7.8	29.9 ± 6.9	30.0 ± 8.8	0.982
LAScd, *%*	17.1 ± 6.4	17.8 ± 6.1	16.4 ± 6.7	0.200
LASct, %	12.8 ± 3.9	12.2 ± 3.2	13.6 ± 4.5	0.019

LVEDD, left ventricular end-diastolic diameter; E/A ratio, peak early mitral inflow velocity / peak late mitral inflow velocity ratio; e’, peak early diastolic mitral annular displacement velocity; TR, tricuspid regurgitation; LAV, left atrial volume; LAV_BSA_, left atrial volume indexed to BSA; LAV_h2_, left atrial volume indexed to height^2^; LASr, left atrial reservoir strain; LAScd, left atrial conduit strain; LASct, left atrial contractile strain. LAE_h2_ was defined as > 16.5 ml/m^2^ for females and > 18.5 ml/m^2^ for males. Normally distributed data are presented as mean ± sd, non-normally distributed data are presented as median (25th interquartile – 75th interquartile), categorical data are presented as n (%). P-value represents comparison between LAE_h2_ and No LAE_h2_

### Comparison between patients with and without LAE_h2_

As presented in Table 1, there was a small but significant difference in age (51.1 ± 7.9 years vs. 53.7 ± 6.2 years, p = 0.033) between the groups. Patients in the LAE_h2_ group more often had a history of hypertension and more often used diuretics (42.9% vs. 24.6%, p = 0.023, and 26.0% vs. 10.8%, p = 0.021, respectively).

Echocardiographic parameters are shown in Table 2. Apart from an expected significant difference in LAV_BSA_ (30.8 ± 6.0 ml/m^2^ vs. 19.6 ± 3.1 ml/m^2^, p < 0.001), there were no differences in other conventional diastolic echocardiographic parameters between the groups. As for LA function, the LAE_h2_ group had significantly lower LASct (12.2 ± 3.2% vs. 13.6 ± 4.5%, p = 0.019). There was no difference in LASr and LAScd between groups.

### LAE in relation to LASct

**Fig. 4:**
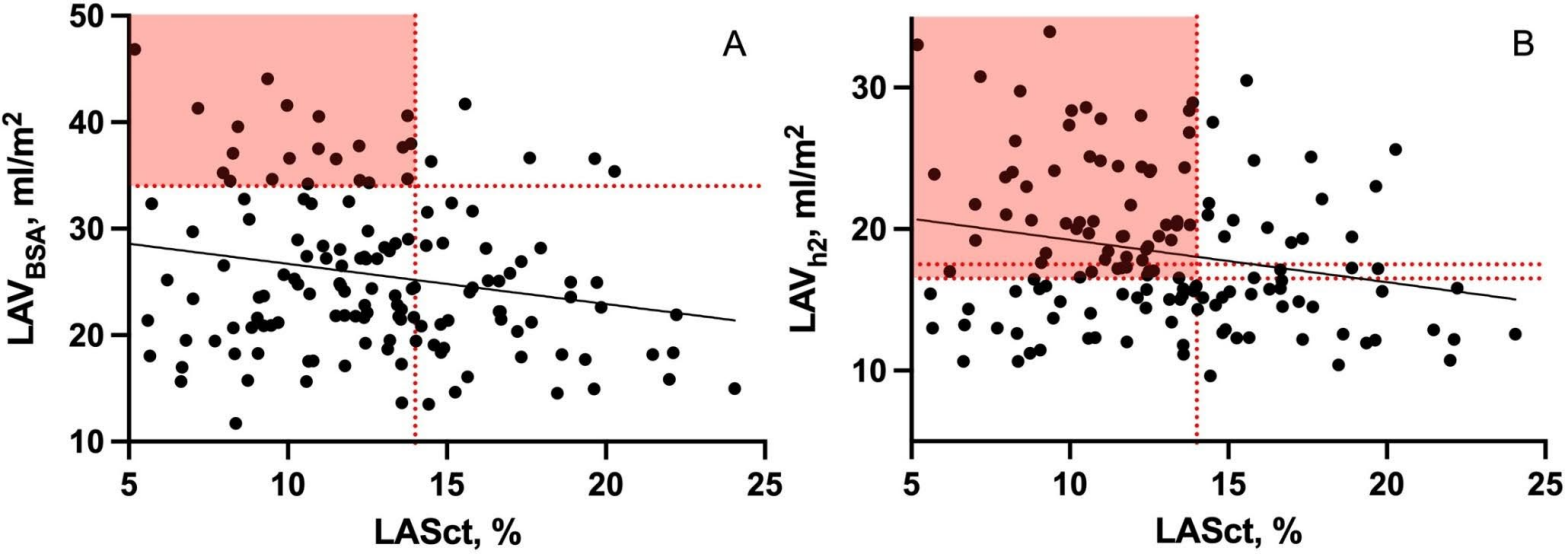
**A**: Relation between left atrial volume indexed to body surface area (LAV_BSA_) and left atrial contractile strain (LASct). Horizontal red dashed line represents left atrial enlargement indexed to body surface area threshold. Vertical red dashed line represents left atrial strain contractile dysfunction. **B**: Relation between left atrial volume indexed to height^2^ (LAV_h2_) and LASct. Horizontal red dashed lines represent left atrial enlargement indexed to height^2^ thresholds (female and male respectively). Vertical red dashed line represents left atrial strain contractile dysfunction

**Fig. 5:**
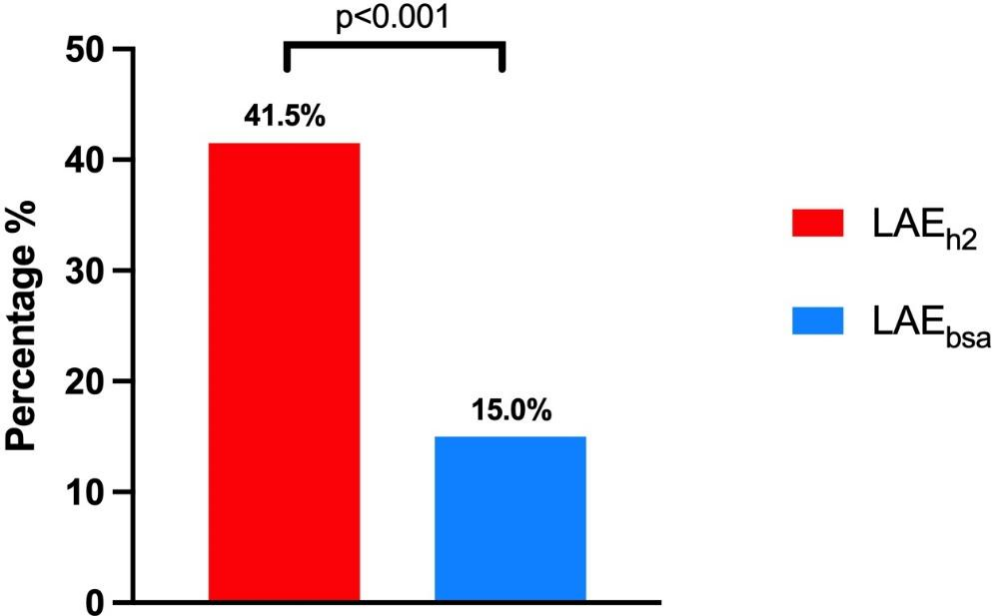
Blue bar represents patients with left atrial dysfunction and left atrial enlargement when left atrial volume was indexed to body surface area (LAE_h2_). Red bar represents patients with left atrial dysfunction and left atrial enlargement when left atrial volume was indexed to height^2^ (LAE_bsa_).

In Fig. 4 the correlations between LAV_h2_, LAV_BSA_, and LASct are depicted. There was a significant, but weak, negative correlation for both LAV_h2_ and LASct (r=-0.22, p = 0.009) and LAV_BSA_ and LASct (r=-0.21, p = 0.015). Significantly more patients with LA dysfunction as defined by LASct < 14% would have been correctly classified by LAE_h2_ as compared to LAE_BSA_ (41.5% vs. 15.0%, p < 0.001) (Figs. 4 and 5). Table 3 shows the association of various LV diastolic parameters with LASct. In binary logistic regression LAE_h2_ was significantly associated with an abnormal LASct (OR 2.64, CI 1.29–5.42, p = 0.008).

**Table 3:** Association of different left ventricular diastolic parameters with left atrial contractile strain

Dichotomous analysis	Abnormal LASct strain	
	OR (95% CI)	p value
Abnormal septal e’ velocity *(< 7 cm/s)*	0.52 (0.26–1.0)	0.067
Abnormal E/e’ average *(> 14)*	0.73 (0.19–2.70)	0.632
LAE_h2,_*ml/m*^*2*^	2.64 (1.29–5.42)	0.008
LAE_BSA,_*ml/m*^*2*^	2.38 (0.84–6.79)	0.104

LASct, left atrial contractile strain; e’, peak early diastolic mitral annular displacement velocity; E, peak early mitral inflow velocity; LAE_h2_, left atrial enlargement indexed to height^2^; LAE_BSA_, left atrial enlargement indexed to body surface area; CI, confidence interval; OR, odds ratio. Abnormal LASct strain was defined as LASct strain < 14%, LAE_BSA_ >34 ml/m^2^, and LAE_h2_ as > 16.5 ml/m^2^ for females and > 18.5 ml/m^2^ for males

## Discussion

We demonstrated that, in subjects with moderate and severe obesity without known cardiac disease, LAE_h2_ was associated with an increased risk for LA dysfunction, in contrast to LAE_BSA_ and other traditional parameters of LV diastolic function. Furthermore, we confirmed findings of previous studies, showing that indexation of LAV to height^2^ resulted in a higher prevalence of LAE compared to indexation of LAV to BSA in these subjects. Considering the limitations of indexation to BSA in obesity, LAE_h2_ may be of added value in determining increased risk of cardiac dysfunction in patients with obesity.

### LAE in obesity

Obesity is an important risk factor for developing LAE [18], which is an essential parameter in identifying diastolic dysfunction and HFpEF [1, 2]. In addition, both obesity and LAE are associated with an increased risk for developing atrial fibrillation (AF) [19–22]. There are several mechanisms by which obesity can lead to LAE. For example, obesity can induce hemodynamic changes that can alter cardiac structures, it can cause atrial myopathy related to systemic inflammation, and promote paracrine effects from epicardial adipose tissue [23–25].

Normalization of heart chamber sizes is common and necessary, as it reduces the effect of dissimilarities in patients’ proportions. Additionally, normalization allows inter- and intragroup comparisons of cardiac dimensions [26]. Normal values enable the possibility to define normal ranges, that can be used to predict, diagnose, and monitor disease. The use of BSA as indexation method in LA scaling dates back to the 1980s [27], and is still recommended in the current guidelines [2]. However, indexation of LAV to BSA is inaccurate for patients with obesity [6]. The reasons for this are several fold. First of all, indexing LAV to BSA assumes a linear relationship. However, data on the growth patterns of the human heart indicate that the growth relationship is exponential rather than linear [26, 28, 29]. This can be overcome by choosing allometric scaling instead, as allometric scaling assumes an exponential relationship [6]. A few previous studies have assessed different indexation methods in patients with obesity. First, Zong et al. found that allometric scaling was superior to conventional isometric indexation in a population of 717 patients with obesity with a mean BMI of 42.2 kg/m2 [30]. Second, in a paper by Carnavelini et al., a similar conclusion was drawn in 63 patients with mild, and 26 patients with moderate obesity [31]. Although both studies demonstrated that allometric scaling was superior to isometric scaling, potential supportive data regarding the relation of alternative indexing methods with LA function was not available.

The second concern with indexing LAV to BSA in obesity, is that cardiac size is driven by fat free mass (FFM) [26]. In normal weight subjects, BSA is a suitable surrogate for FFM and thus a suitable scaler to index LAV [32]. However, in patients with obesity, BSA is disproportional to FFM and therefore possibly overcorrects LAV [6]. Height appears to be a better estimate for FFM [6]. Our results are consistent with this notion, as can be seen in Fig. 1 where LAV indexed to height^2^ was related to increasing BMI as expected, in contrast to LAV indexed to BSA. In addition, we found that indexing LAV to height^2^ resulted in a higher prevalence of LAE compared to BSA. A recent study showed similar results, where as many as 55.4% of the severely obese patients were reclassified as having LAE when height^2^ was used for indexation instead of BSA [7]. Additionally, recent studies have demonstrated that indexation of LAV to height^2^ has better predictive value concerning clinical outcomes in patients with obesity [7, 8]. However, both studies did not investigate the relation between LAV_h2_ and LA function.

### Relation between LAV_h2_ and LA function in obesity

In order to investigate whether LAV_h2_ may also better identify LA dysfunction in patients with obesity as compared to LAV_BSA_, this study was the first to relate LAV_h2_ and LAV_BSA_ to LA strain. Recently, LA strain has emerged as a parameter that has potential added value in identifying diastolic dysfunction. LASr and LASct are both associated with LV filling pressures [33–35]. Patients with obesity with LAE_h2_ had significantly lower LASct compared to patients without LAE_h2_. Also, more patients with abnormal LASct were identified by LAE_h2_ as compared to LAE_BSA_. In addition, LAE_h2_ was associated with an increased risk (OR 2.64) for an abnormal LASct, in contrast to LAE_BSA_ and other traditional diastolic parameters. Our novel findings underscore the notion that LAV_h2_ is not only a more sensitive measure of LAE in patients with obesity, but indeed more sensitive for identification of LA dysfunction as well. As LAE and LA dysfunction are important parameters of LV diastolic dysfunction, use of LAV_h2_ may improve the utility of a diastolic function qualification algorithm. However, we have not investigated that in our study. Further studies confirming improved prognostic value of LAV_h2_ as compared to LAV_BSA_ are mandatory first.

### Study limitations

This study has some limitations that should be noted. First of all, LA strain analysis requires good image quality and not all our subjects (26%) had analyzable LA images, which may have affected the identified proportion of LA dysfunction. Second, a considerable proportion of the subjects had comorbidities, such as hypertension and diabetes, that can also affect LA function. Third, our cohort mostly consisted of females which could have biased the results. Around 80% of patients who undergo bariatric surgery are female, which explains the high percentage of females in our study. Fourth, only LASct and not LASr and LAScd were different between patients with and without LAE_h2_. Although most of the previous research has focused on LASr, added value of LASct has already been proven as well [16] and is therefore also considered to be an important measure of LA function. Finally, diagnostic value of other echocardiographic parameters in subjects with moderate to severe obesity may also improve when indexed to height^2^ instead of BSA. However, this fell beyond the scope of the current study.

## Conclusion

Relatively easy assessment of LAV_h2_ could overcome inherent limitations of LAV_BSA_ in patients with obesity and thereby contribute to the detection of cardiac dysfunction in these patients. LAV_h2_ was more sensitive for detection of LAE and better related to LA dysfunction as compared to the current standard of normalization of LAV for BSA in our population of patients with moderate to severe obesity without known cardiac disease. With the rising prevalence of obesity worldwide, it is pivotal to have an early and accurate assessment of cardiac dysfunction in order to prevent further deterioration to heart failure. Early detection can lead to timely initiation of lifestyle modifications and treatment, and therefore reduce the associated risks and morbidity of obesity.
